# Implementation of a COVID-19 surveillance programme for healthcare workers in a teaching hospital in an upper-middle-income country

**DOI:** 10.1371/journal.pone.0249394

**Published:** 2021-04-14

**Authors:** Kim Sui Wan, Peter Seah Keng Tok, Kishwen Kanna Yoga Ratnam, Nuraini Aziz, Marzuki Isahak, Rafdzah Ahmad Zaki, Nik Daliana Nik Farid, Noran Naqiah Hairi, Sanjay Rampal, Chiu-Wan Ng, Mohd Fauzy Samsudin, Vinura Venugopal, Mohammad Asyraf, Narisa Hatun Damanhuri, Sanpagavalli Doraimuthu, Catherine Thamarai Arumugam, Thaneswaran Marthammuthu, Fathhullah Azmie Nawawi, Faiz Baharudin, Diane Woei Quan Chong, Vivek Jason Jayaraj, Venna Magarita, Sasheela Ponnampalavanar, Nazirah Hasnan, Adeeba Kamarulzaman, Mas Ayu Said

**Affiliations:** 1 Centre for Epidemiology and Evidence-Based Practice, Department of Social and Preventive Medicine, Faculty of Medicine, University of Malaya, Kuala Lumpur, Malaysia; 2 Ministry of Health Malaysia, Putrajaya, Malaysia; 3 University Malaya Medical Centre, Kuala Lumpur, Malaysia; 4 Department of Medicine, Faculty of Medicine, University of Malaya, Kuala Lumpur, Malaysia; 5 Department of Rehabilitation Medicine, Faculty of Medicine, University of Malaya, Kuala Lumpur, Malaysia; University of Auckland, NEW ZEALAND

## Abstract

**Introduction:**

The reporting of Coronavirus Disease 19 (COVID-19) mortality among healthcare workers highlights their vulnerability in managing the COVID-19 pandemic. Some low- and middle-income countries have highlighted the challenges with COVID-19 testing, such as inadequate capacity, untrained laboratory personnel, and inadequate funding. This article describes the components and implementation of a healthcare worker surveillance programme in a designated COVID-19 teaching hospital in Malaysia. In addition, the distribution and characteristics of healthcare workers placed under surveillance are described.

**Material and methods:**

A COVID-19 healthcare worker surveillance programme was implemented in University Malaya Medical Centre. The programme involved four teams: contact tracing, risk assessment, surveillance and outbreak investigation. Daily symptom surveillance was conducted over fourteen days for healthcare workers who were assessed to have low-, moderate- and high-risk of contracting COVID-19. A cross-sectional analysis was conducted for data collected over 24 weeks, from the 6^th^ of March 2020 to the 20^th^ of August 2020.

**Results:**

A total of 1,174 healthcare workers were placed under surveillance. The majority were females (71.6%), aged between 25 and 34 years old (64.7%), were nursing staff (46.9%) and had no comorbidities (88.8%). A total of 70.9% were categorised as low-risk, 25.7% were moderate-risk, and 3.4% were at high risk of contracting COVID-19. One-third (35.2%) were symptomatic, with the sore throat (23.6%), cough (19.8%) and fever (5.0%) being the most commonly reported symptoms. A total of 17 healthcare workers tested positive for COVID-19, with a prevalence of 0.3% among all the healthcare workers. Risk category and presence of symptoms were associated with a positive COVID-19 test (p<0.001). Fever (p<0.001), cough (p = 0.003), shortness of breath (p = 0.015) and sore throat (p = 0.002) were associated with case positivity.

**Conclusion:**

COVID-19 symptom surveillance and risk-based assessment have merits to be included in a healthcare worker surveillance programme to safeguard the health of the workforce.

## Introduction

Since its first description in December 2019, the novel coronavirus SARS-CoV-2 has caused a worldwide pandemic of Coronavirus Disease 19 (COVID-19) with 71,581,532 confirmed cases and 1,618,374 deaths worldwide as of 15^th^ of December 2020 [1]. The highly transmissible nature of SARS-CoV-2 has facilitated the clustering of transmission in congregational settings such as nursing homes, detention facilities, and hospitals [2]. From the beginning of this epidemic in China, the high risk of infection to healthcare workers (HCWs) and intra-hospital transmission of the virus to staff and patients were recognised, with various measures taken to minimise the infection [3, 4].

As of June 2020, a total of 152,888 infections and 1,413 deaths among HCWs have been reported [5]. The mortality among HCWs has been reported in hardest-hit countries such as China (23 deaths as of 3^rd^ of April 2020), Italy (105 deaths as of 5^th^ of April 2020), and the UK (157 deaths as of 3^rd^ of May 2020) [6–9]. During this ongoing global crisis, HCWs is estimated to constitute 7% of all COVID-19 cases globally [10]. Growing HCW infections will lead to reductions in the healthcare capacity, which is central in ensuring the pandemic does not overwhelm a country’s healthcare system. This has led healthcare organisations to implement strategies that range from traditional occupational surveillance programs to more adaptive and higher resource strategies such as periodic or intermittent testing in order to protect their HCWs [11]. These approaches should be adapted based on the local epidemiology of disease and resource availability [11]. In low- and middle-income countries, major challenges with COVID-19 testing were inadequate capacity, untrained laboratory personnel, and inadequate funding, calling for alternative strategies such as clinical-based triaging and more prudent use of resources [12, 13].

Malaysia, an upper-middle-income country, reported its first COVID-19 patient on the 24^th^ of January 2020 [14]. Initially, the reported local transmission remained relatively low, as cases were primarily imported. However, a sharp increase in cases occurred in early March following an outbreak among members of a large religious gathering. This prompted the government to declare the situation as a national security concern, which led to the implementation of a nationwide lockdown, locally known as the Movement Control Order (MCO). Central to this national control policy has been the mandate to admit all cases to COVID-19 hospitals for isolation and treatment, further amplifying the need to protect HCWs [15]. As of mid-May 2020, 5.8% of infections in Malaysia occurred among HCWs [16].

Following its designation as a COVID-19 hospital in Malaysia, the University Malaya Medical Centre (UMMC) COVID-19 Task Force conceived a comprehensive plan in ensuring the safety of its healthcare workforce while ensuring continuity of service. The strategies include a) cohorting COVID-19 patients to designated wards, b) development of clinical pathways and guidelines for the management of patients, c) ensuring adequate supply and adherence to PPE usage, d) extensive training of the healthcare workforce, and v) the development of a HCW surveillance programme.

The HCW surveillance programme, in particular, leveraged a risk-based testing and isolating strategy that allowed for surveillance while balancing the use of resources. We aim to describe the components and implementation of the HCW surveillance programme in UMMC. In addition, we describe the distribution and characteristics of the HCWs placed under surveillance.

## Methodology

### Study design and setting

This was a cross-sectional analysis of a HCW surveillance programme in UMMC over 24 weeks from the 6^th^ of March 2020 to the 20^th^ of August 2020. Adherence to the surveillance programme by all HCWs is sanctioned as an administrative policy by UMMC to ensure the safety of its workforce. UMMC is a teaching hospital located in Kuala Lumpur and was one of the sixty hospitals designated to manage COVID-19 cases in Malaysia. UMMC has a total of 1,600 beds that caters for paediatric and adult patients, with a comprehensive range of speciality areas. Following its designation as a COVID-19 hospital, 153 beds across eight wards were allocated for COVID-19 patients, with 22 being Intensive Care Unit (ICU) beds. The hospital employed 5,826 HCWs, of which 1,987 staff were assigned to care exclusively for COVID-19 cases.

### Ethical consideration

Ethical approval and waiver of written informed consent were obtained from the UMMC Medical Research Ethics Committee (MECID. No 202073–8862). The permission to collate data was obtained from the UMMC COVID-19 Taskforce. Dataset retrieved for this analysis did not contain any personal information, and there was no interaction with any HCWs. HCWs were not intentionally put at risk of exposure for the purpose of this study; they were existing professionals contracted to their routine work obligations at UMMC. There is standard practice for infection control for which HCWs have been trained, and appropriate PPE was provided.

### The UMMC COVID-19 operations room

**Fig 1:**
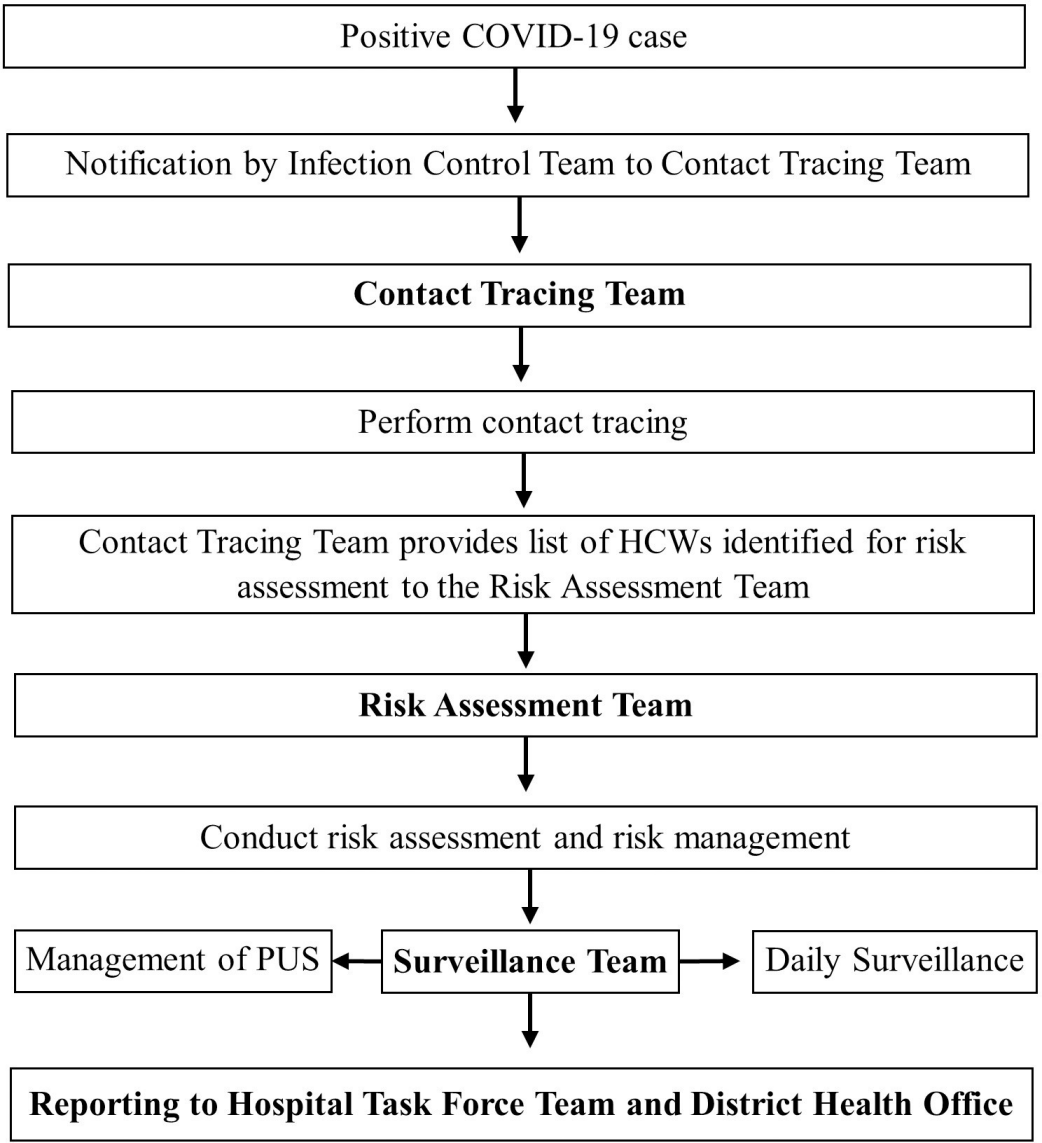
General workflow at the UMMC COVID-19 operations room. *PUS, Person Under Surveillance.

The COVID-19 Operations Room, also known as a hospital emergency operation centre, was set up to prevent and manage COVID-19 outbreaks among UMMC HCWs through activities that focused on early detection and disease containment. The operations room workforce was categorised into four work teams, namely contact tracing, risk assessment, surveillance, and outbreak investigation. The flow of events following the identification of a COVID-19 positive case is illustrated in **Fig 1** [17].

The hospital’s Infectious Disease team notified the Contact Tracing team whenever a new COVID-19 case was identified. A new COVID-19 case was either an existing inpatient who was newly diagnosed with COVID-19, a new admission diagnosed with COVID-19 referred to UMMC, or a HCW from UMMC infected with COVID-19. The activation of the contact tracing team would initiate a cascade of activities to gather and collate information pertaining to the movement of the infected person 48 hours before the diagnosis or the onset of symptoms. HCWs who had been in contact with the infected person would be listed as new contacts, while the non-HCW contacts were managed by personnel from respective District Health Offices (DHO) under the Ministry of Health (MOH).

The list of new contacts was then relayed to the Risk Assessment team. The level of risk of the new contacts was assessed and stratified according to their risk level based on the “assessment checklist for healthcare workers” **(S1 Appendix).** The contacts were then classified into four different risk categories, namely ’no identifiable risk’, ’low risk’, ’medium risk’ or ’high risk’, based on the UMMC guidelines [17]. The UMMC COVID-19 surveillance guidelines for healthcare worker was consistent with the Ministry of Health guidelines [15]. A ’low risk’ classification referred to HCWs who had close contact with a positive COVID-19 case while using the recommended Personal Protective Equipment (PPE) [17]. A ’medium risk’ referred to HCWs who had prolonged close contact with COVID-19 cases without the proper use of PPE (breach of PPE). Prolonged close contact referred to exposure to a COVID-19 case for more than 15 minutes and within proximity of two meters or lesser [17]. The ’high risk’ group referred to HCWs who were not protected by PPE while performing aerosol-generating procedures (AGP) or were in any other circumstances in which exposure to respiratory secretions was likely [17]. The recommended set of PPEs for different types of activities or procedures are shown in the **S2 Appendix**. For example, face shield, N95 mask, gloves, coverall suit, shoe cover and proper hand hygiene are recommended for aerosol-generating procedures. Detailed management algorithm of HCWs in high-, medium-, and low-risk groups are shown in **S3–S5 Appendices**. Nasopharyngeal swabs for RT-PCR were taken based on the level of risk and presence of symptoms. Those categorised as ’no identifiable risk’ were not subjected to work restrictions nor placed under active surveillance.

In the next stage, HCWs classified as low, moderate, and high risk were placed under active surveillance and designated as ’Person Under Surveillance’ (PUS). Their symptoms were monitored for 14 days from the date of their last exposure to a COVID-19 case by the surveillance team. A PUS who developed symptom(s) during the surveillance period was immediately referred to the Risk Assessment team for further management.

### Data collection

All epidemiological data were collected from the COVID-19 operation room’s centralised electronic database, which served as a digital repository for HCWs contact tracing, risk assessment and surveillance records. Additional retrievable data included sociodemographic information such as age, sex, comorbidities, and designation of the HCWs. All captured data were stored in a password-protected database which was only accessible to a few authorised personnel [18].

The exposure variables in this analysis were age, sex, job category, presence of comorbidities, risk category, and symptoms during surveillance. The symptoms, namely fever, cough, dyspnoea, sore throat, arthralgia, myalgia, gastrointestinal (GI) symptoms, and anosmia were self-reported. The symptom surveillance for anosmia was only started on the 4^th^ of April 2020. The outcome variable was HCWs with COVID-19, defined as a person with a laboratory confirmation for SARS-CoV-2 by reverse transcriptase-polymerase chain reaction (RT- PCR) [15].

### Phases of surveillance

During the initial phase, the surveillance team used an instant messaging system (WhatsApp application) to disseminate a standardised daily symptoms surveillance message to all PUS. This message enquired on any new symptom(s) for the day, any worsening of symptoms and the date of last exposure to COVID-19 patients. The selection of symptoms listed were fever, cough, breathing difficulty, myalgia, arthralgia, and any gastrointestinal symptom (anosmia was later added to the list from the 4^th^ of April 2020 onwards). The PUS would indicate if any symptoms were present and the type of symptoms (if any). Those who did not reply by afternoon each day were contacted by the surveillance team via text messaging or a phone call.

The number of PUS by the 31^st^ of March 2020 was 475, reflecting a rapid rise since the operation first began with only 10 PUS on the 26^th^ of February 2020. During the second phase, a dedicated web-based surveillance system was co-designed and created by the Operation Room workforce and the Department of Information Technology. This self-reporting surveillance tool was integrated with the existing UMMC staff portal system. Data that were keyed into the digital surveillance sheet and submitted via the portal were readily retrievable by the surveillance team through a secured backend login page. This data interoperability between user and backend interfaces permitted auto-generation of daily surveillance reports and alert notifications of PUS whose scores were categorised as moderate or high risk. **S6 Appendix** provides more information about the UMMC portal for COVID-19 healthcare workers risk assessment and surveillance.

With this new approach, a PUS would receive the standard symptoms surveillance message in the form of an automated Short Messaging Service (SMS). The surveillance text message contained a link directing them to an online feedback form in the UMMC staff portal, and their responses were recorded in the database. Surveillance messages were sent at 6 am daily to elicit feedback on their general wellbeing. In the event that a PUS failed to reply, a second message was then sent at 10 am and a final reminder at 12 pm. Any PUS who failed to respond was contacted by the surveillance team via text messages or phone call.

### Statistical analyses

Descriptive analysis was performed for the baseline characteristics. Pearson chi-square tests were used to compare the proportions of HCWs with and without COVID-19; Fischer’s exact tests were conducted when more than 20% of the cells had expected frequencies below five [19]. Non-parametric Mann-Whitney test was carried out to compare the median age. Statistical significance was pre-set at *P* <0.05. The prevalence of COVID-19 was calculated as the proportion of HCWs diagnosed as per case definition over the total number of staff in UMMC.

A post hoc analysis was conducted to calculate the sensitivity and specificity of the risk-based assessment in identifying cases and non-cases. All analyses were conducted using the IBM SPSS version 23 (IBM Corporation, Armonk, NY, USA).

## Results

**Fig 2:**
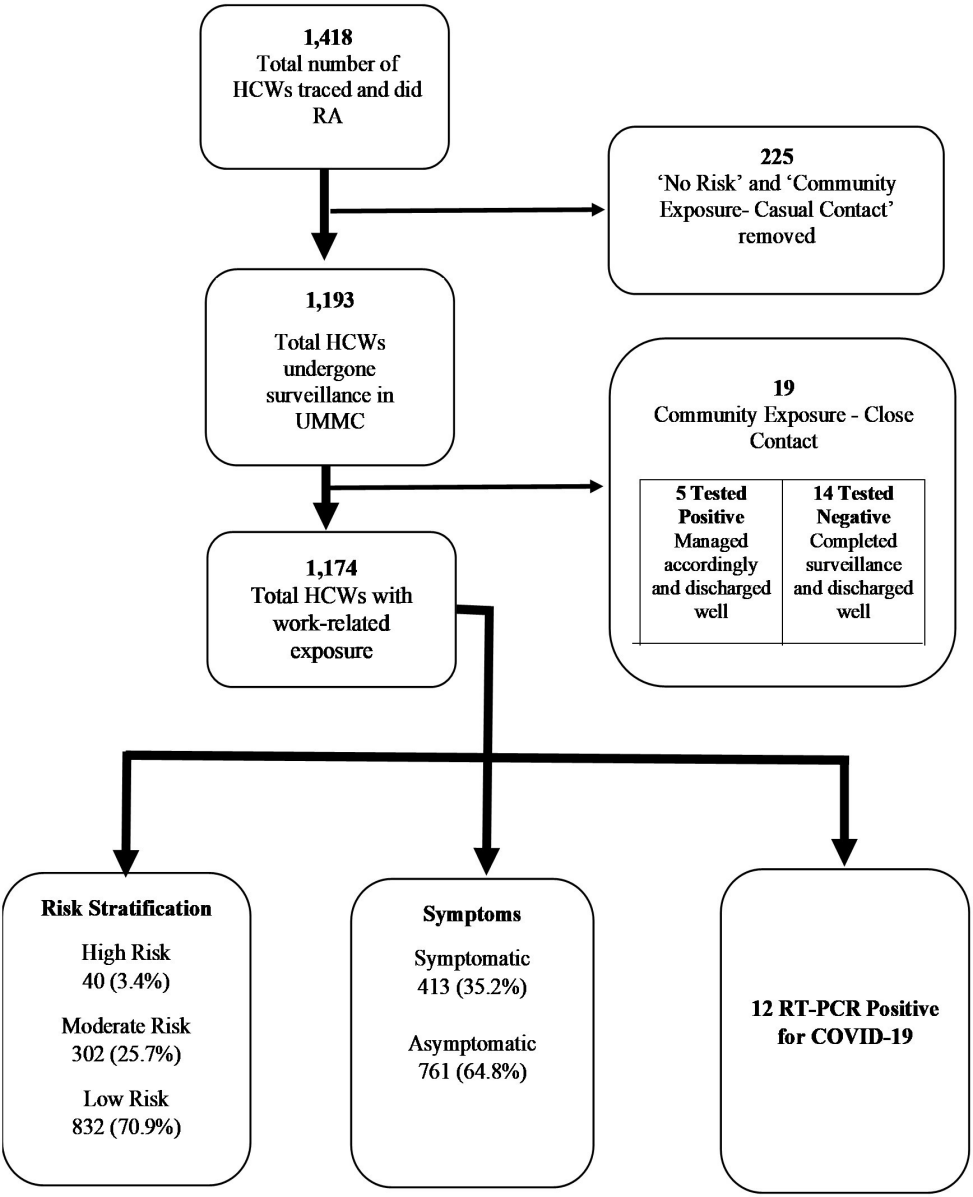
Surveillance flow chart and total HCWs for the study. The surveillance system was only meant for HCWs with work-related exposure. HCWs with community exposure were under the surveillance of the District Health Office. *HCW, healthcare worker; UMMC, University Malaya Medical Centre; RT-PCR, reverse transcriptase-polymerase chain reaction.

The surveillance flowchart, along with the total number of HCWs included in the study, is detailed in **Fig 2**. From a total of 5,826 HCWs in UMMC during the study period, almost a quarter of them (n = 1,418; 24.3%) were exposed to COVID-19 infection and had their risk assessed. After excluding HCWs who had ‘no identifiable risk’ and ‘community exposure with casual contact’, a total of 1,193 HCWs were placed under surveillance, of which 1,174 HCWs had work-related exposure. All 1,174 HCWs who had work-related exposure were included for analysis.

Baseline characteristics and surveillance outputs for all HCWs who had work-related exposure (n = 1,174) are presented in **Tables 1** and **2**, respectively. Throughout the study period, a total of 17 HCWs (12 HCWs had work-related exposure and 5 HCWs had community exposure–close contact) tested positive for COVID-19. This translates into a prevalence of 0.3% (17 out of 5,826) COVID-19 case positivity among the HCW population in UMMC. As soon as each of these HCWs were tested positive, isolation measures were initiated, which constituted a 14-day admission to a COVID-19 designated ward regardless of the presence or absence of symptoms. Following these actions, no onward transmissions were recorded from infected HCWs. All HCWs who were placed under the surveillance program completed the stipulated surveillance duration. The mean daily response rate (number of PUS responded over total daily PUS) was 96.3% (95% CI: 95.6–97.0) with a range from 88% to 100%.

**Table 1:** Baseline characteristics for all HCW who had work-related exposure (n = 1,174).

Baseline characteristics			n	%
**Age in years, median (IQR)**			30 (8)	
	Minimum 18 years old, maximum 60 years old			
**Age groups**				
	24 years and below		157	13.4
	25 to 34 years old		759	64.6
	35 to 44 years old		204	17.4
	45 to 54 years old		48	4.1
	55 years old and above		6	0.5
**Sex**				
	Male		333	28.4
	Female		841	71.6
**Job category**				
	Medical doctor		325	27.7
	Nursing		551	46.9
	Allied health		259	22.1
	Ancillary personnel		39	3.3
**Comorbidities**				
	No		1,042	88.8
	Yes		132	11.2
		Bronchial asthma	45	3.8
		Rhinitis and/or sinusitis	26	2.2
		Hypertension	20	1.7
		Diabetes mellitus	12	1.0
		Tuberculosis	6	0.5
		Others	29	2.5

Cumulative total for all comorbidities may exceed total n = 1,174 as there might be HCWs with multiple comorbidities.

**Table 2:** Surveillance variables for all HCWs who had work-related exposure (n = 1,174).

Characteristic			n	%
**Risk Assessment (work-related exposure)**				
	High Risk		40	3.4
	Moderate Risk		302	25.7
	Low Risk		832	70.9
**Symptoms during surveillance**				
	No–Asymptomatic		761	64.8
	Yes—Symptomatic (at least one symptom)		413	35.2
		Fever	59	5.0
		Cough	232	19.8
		Dyspnoea	19	1.6
		Sore throat	277	23.6
		Arthralgia	21	1.8
		Myalgia	48	4.1
		Gastrointestinal symptoms	40	3.4
		Anosmia*	5	0.4
		Others	24	2.0

Cumulative total for all symptoms may exceed total n = 1,174 as there might be HCWs with multiple symptoms.

Symptoms under surveillance—took into account presence of any of the symptom(s) stated in the list above.

*Symptom surveillance for anosmia had only started since the 4^th^ of April 2020.

**Table 3** shows the univariable analysis for a potential association between variables of interest and case positivity of HCWs for COVID-19. There was no significant association between HCWs baseline characteristics and the positive COVID-19 test. The risk of exposure, however, was shown to be associated with positive COVID-19 tests (p<0.001). All of the 12 HCWs with work-related exposure who tested positive had at least moderate risk of exposure. Similarly, HCWs having symptoms were significantly associated with a positive COVID-19 test (p<0.001). When specific symptoms were investigated further, fever (p<0.001) and respiratory symptoms–cough (p = 0.003), shortness of breath (p = 0.015) and sore throat (p = 0.002) were found to be significantly associated with case positivity while other symptoms such as arthralgia, myalgia, gastrointestinal symptoms, and anosmia had no significant association.

**Table 3:** Univariable analysis for baseline characteristics and surveillance variables (n = 1,174).

Variable		All	HCW with COVID-19	All other HCW	*P* values
		(n = 1,174)	(n = 12)	(n = 1,162)	
**Age, in years**		** **			0.400†
	Median (IQR)	30.0 (8.0)	28.5 (8.0)	30.0 (8.0)	
**Age groups**		** **			0.898*
	24 years old and below	157	1 (0.6)	156 (99.4)	
	25 to 34 years old	759	8 (1.1)	751 (98.9)	
	35 to 44 years old	204	3 (1.5)	201 (98.5)	
	45 to 54 years old	48	0 (0.0)	48 (100.0)	
	55 years old and above	6	0 (0.0)	6 (100.0)	
**Sex**		** **			0.749*
	Male	333	4 (1.2)	329 (98.8)	
	Female	841	8 (1.0)	833 (99.0)	
**Job position/category**		** **			0.509*
	Medical	325	2 (0.6)	323 (99.4)	
	Nursing	551	6 (1.1)	545 (98.9)	
	Allied health	259	3 (1.2)	256 (98.8)	
	Ancillary personnel	39	1 (2.6)	38 (97.4)	
**Comorbidities**					0.635*
	Yes	132	2 (1.5)	130 (98.5)	
	No	1,042	10 (1.0)	1,032 (99.0)	
**Risk Assessment**					<0.001*
	High Risk	40	5 (12.5)	35 (87.5)	
	Moderate Risk	302	7 (2.3)	295 (97.7)	
	Low Risk	832	0 (0.0)	832 (100.0)	
**Symptoms during surveillance**					0.001*
	Yes—Symptomatic	413	10 (2.4)	403 (97.6)	
	No—Asymptomatic	761	2 (0.3)	759 (99.7)	
**Fever**					<0.001*
	Yes	59	6 (10.2)	53 (89.8)	
	No	1,115	6 (0.5)	1,109 (99.5)	
**Cough**					0.003*
	Yes	232	7 (3.0)	225 (97.0)	
	No	942	5 (0.5)	937 (99.5)	
**Dyspnoea**					0.015*
	Yes	19	2 (10.5)	17 (89.5)	
	No	1,155	10 (0.9)	1,145 (99.1)	
**Sore throat**					0.002*
	Yes	277	8 (2.9)	269 (97.1)	
	No	897	4 (0.4)	893 (99.6)	
**Arthralgia**					0.196*
	Yes	21	1 (4.8)	20 (95.2)	
	No	1,153	11 (1.0)	1,142 (99.0)	
**Myalgia**					0.396*
	Yes	48	1 (2.1)	47 (97.9)	
	No	1,126	11 (1.0)	1,115 (99.0)	
**Gastrointestinal symptoms**					0.342*
	Yes	40	1 (2.5)	39 (97.5)	
	No	1,134	11 (1.0)	1,123 (99.0)	
Anosmia*					1.000*
	Yes	5	0 (0.0)	5 (100.0)	
	No	1,169	12 (1.0)	1,157 (99.0)	

† Mann-U Whitney test

* Fisher’s Exact test. Symptom surveillance for anosmia had only started since the 4^th^ of April 2020.

In the post hoc analysis, the risk-based assessment employed in this surveillance was found to have a sensitivity approaching 100% in identifying positive COVID-19 cases **(Table 4).** The ability to correctly identify HCWs who did not have COVID-19 or the specificity was around 72%. It should be noted, however, that not all HCWs in the low-risk category were tested. From the 832 HCWs in the low-risk category, only 282 HCWs (33.9%) were tested, with all of them yielding negative results. After excluding HCWs who were not tested, the specificity of this risk-based assessment was 46.1%. (= 282/612). Thus, the result needs to be interpreted cautiously.

**Table 4:** Cross-tabulation of risk category and RT-PCR results (n = 1,174).

Risk category	Swab RT-PCR Results		
	Positive n (%)	All other HCW n (%)	Total n (%)
High or moderate	12 (100.0)	330 (28.4)	342 (29.1)
Low	0 (0.0)	832 (71.6)	832 (70.9)
Total	12 (100.0)	1,162 (100.0)	1,174 (100.0)

## Discussion

In this article, we describe how UMMC, a teaching hospital in an upper-middle-income country, adapted a surveillance system to safeguard the welfare of its HCWs against the COVID-19 pandemic. The HCW surveillance programme leveraged a risk-based testing and isolating strategy which do not require high resources for its operation.

The establishment of the Operations Room represents the cornerstone of UMMCs COVID-19 prevention, containment and mitigation strategy. Throughout the 24 weeks, teams running the operations room tried to optimise the HCW surveillance system. The technological upgrade from the WhatsApp application (Phase 1) to the online portal-based surveillance system (Phase 2) was necessary to respond to the increasing number of PUS and in anticipation of the additional workload that ensued. This was because Phase 1 of the surveillance system required manual data entry that was time-consuming and laborious. Therefore, a partially automated mechanism, i.e. Phase 2 of the surveillance system, was developed by digitalising and integrating the surveillance processes into the existing UMMC portal for HCWs. This system allowed responses from the PUS to be gathered and analysed, producing a more streamlined workflow and organised database. Furthermore, the evolution from Phase 1 to Phase 2 not only improved the efficiency and sustainability of the surveillance system but was also resource-effective.

The shift from a manual to an online reporting mechanism was met with some resistance from a small number of HCWs in the early stages. We received feedback that the portal-based surveillance monitoring was tedious as it required the PUS to log onto the UMMC portal and fill up the surveillance reporting form on their own. In order to promote acceptability and compliance, continuous education and training were done to familiarise users with the portal, keeping non-respondent rates at a minimal number. We also ensured that all PUS were given an adequate explanation on how and why the surveillance was done [20]. These actions produced good adherence in reporting of symptoms among PUS.

Every PUS who failed to respond to the daily surveillance messages was contacted. For PUS who did not respond for three consecutive days, their names were submitted to the hospital management for further action. The PUS compliance was further enhanced by email reminders from the Hospital Director. The effort and resources put into developing this surveillance system bridged a service gap in a time of need and serves as an investment for the future.

### Characteristics of HCWs and prevalence of COVID-19

In this study, the characteristics of the HCWs under surveillance was comparable to a study done in Wuhan whereby the younger aged population, females and also nurses predominated [21]. The relatively younger aged HCWs with lesser comorbidities may have contributed to the zero mortality [22]. The COVID-19 prevalence of 0.3% among the entire HCWs population in UMMC contrasted with the 1.0–1.1 percentage in hospitals based in Wuhan and Netherlands [21, 23]. Our relatively lower prevalence could be attributed to the fact that Wuhan was the epicentre of the COVID-19 pandemic [24]. Similarly, the overall rate of COVID-19 in the Netherlands was much higher than in Malaysia [25].

### Symptom surveillance

It is important to note the significant heterogeneity of symptoms experienced by the COVID-19 positive HCWs in different countries [21, 23, 26, 27]. In China, it was found that a large proportion of HCWs who tested positive for COVID-19 were symptomatic, with fever being the commonest [21]. Meanwhile, symptom surveillance in UMMC revealed that fever was the third commonest symptom after cough and sore throat. Studies in China and Italy reported more non-respiratory symptoms such as lethargy, myalgia, ageusia, anosmia, and asthenia in comparison to respiratory symptoms like cough, shortness of breath and sore throat [21, 26]. On the other hand, other studies found that apart from fever, respiratory symptoms were more commonly reported than non-respiratory symptoms among HCWs who tested positive for COVID-19, which is similar to our findings [23, 27]. It should be noted as well that our study findings should be interpreted within the context of the symptoms we had monitored for, as they may not be similar to symptoms monitored in other studies or populations. With regards to this, we acknowledge that our study findings may be under-powered for a detailed assessment of symptoms, particularly the less common ones.

### Risk-based assessment

The unique characteristic of our risk-based assessment is that the assignment of risk categories during the risk assessment is not dependent on the symptoms. This is important as there is growing evidence on asymptomatic and pre-symptomatic infection of COVID-19 [22]. Our risk-based assessment has good sensitivity and acceptable specificity. However, it should be noted that the criteria for testing varied between levels of risk. For example, only one-third of low-risk individuals were tested, hence the variation in calculated specificities. Nevertheless, it was reassuring that all of them yielded negative test results.

Given its feasibility and practicality, this risk-based assessment may be suitable to be implemented in healthcare settings with high numbers of COVID-19 cases and limited resources. Other low- and middle-income countries may benefit from our risk-based assessment as challenges with COVID-19 testing such as inadequate capacity, untrained laboratory personnel, and inadequate funding have been highlighted [12, 13]. While we acknowledge that the Centers for Disease Control and Prevention (CDC) have recommended testing of asymptomatic HCWs without known or suspected exposure to SARS-CoV-2 as part of the expanded screening, such practice is not suitable for healthcare settings with limited resources [28].

The decision to prescribe home surveillance or no work restriction is dependent on HCWs’ risks. For example, HCWs with moderate and high-risk were put on home surveillance for 7 and 14 days, respectively, with specific protocol on testing for COVID-19. On the other hand, low-risk asymptomatic HCWs had no work restriction. The increased demand for workforce during this pandemic has posed challenges in the United Kingdom, with retired doctors being asked to consider returning to work and clinical staffs redeployed to areas of greatest need [29]. During the pandemic, the obligation to continue serving may give rise to presenteeism, potentially propagating the COVID-19 transmission among at-risk HCWs [30]. During a COVID-19 outbreak in a hospital in Singapore, it was found that a large number of HCWs with mild symptoms of acute respiratory illness had not sought medical evaluation; thus, the surveillance was unable to detect the cluster in real-time [31]. HCWs who continue to be at work without proper risk assessment gives way for worsening of COVID-19 spread in the hospital, threatening the functionality of the healthcare facility. However, unnecessary home surveillance can also place additional strain on manpower. Therefore, we postulate that having a proactive symptom surveillance mechanism and a stratified risk-based assessment may help retain a sizeable workforce whilst safeguarding HCW’s safety and health.

### Strengths and limitations

We have progressively developed a functional HCW surveillance system since the first COVID-19 case was admitted and made further modifications as the number of cases and new evidence surfaced. Besides that, we described the implementation of a HCW surveillance program and the surrounding issues and outputs that can be used to strengthen the program. Our study findings may potentially benefit other healthcare facilities where the resource for expanded screening is limited.

As in any surveillance system, cases can be missed. Due to the risk-based assessment practice in our surveillance system, asymptomatic cases may have been missed, particularly for HCWs in the low-risk category. Airborne transmission of SARS-CoV-2 can occur under special circumstances, further raising the possibility of undiagnosed cases in our study [32]. If testing becomes more affordable and accessible in the future, the development of an expanded testing protocol to enhance the surveillance system may be necessary. We also acknowledge that data retrieved in the initial phase of our study could be limited due to the manual nature of data entering. However, the extent of this is likely minimal with the presence of an audit team to ensure data completeness. It was also not long after that we shifted to a digital reporting system.

## Conclusion

The value of a well-designed surveillance programme and the importance to innovate methods in tandem with evolving situations is described in our article. The combination of contact tracing, risk assessment and symptom surveillance activities proved to be effective in containing the transmission of COVID-19 among HCWs while optimising the use of resources. Findings from our surveillance program suggest that risk-based assessment and symptom surveillance were associated with COVID-19 positivity, highlighting their roles and importance in a COVID-19 surveillance program to safeguard the health of the workforce in an upper-middle-income country.

## Supporting information

S1 Appendix Assessment checklist for healthcare workers (HCW) who were exposed to COVID-19 patients. (DOCX)

S2 Appendix PPE recommendations. (PDF)

S3 Appendix Management of exposed HCW categorised as high-risk. (DOCX)

S4 Appendix Management of exposed HCW categorised as medium-risk. (DOCX)

S5 Appendix Management of exposed HCW categorized as low-risk. (DOCX)

S6 Appendix UMMC portal for COVID-19 healthcare worker risk assessment and surveillance. (PDF)

## References

[1] World Health Organization. WHO Coronavirus Disease (COVID-19) Dashboard 2020 [updated 15 December 2020; cited on 16 December 2020]. Available from: https://covid19.who.int/.

[2] Leclerc QJ, Fuller NM, Knight LE, Group CC-W, Funk S, Knight GM. What settings have been linked to SARS-CoV-2 transmission clusters? Wellcome Open Res. 2020;5:83. https://dx.doi.org/10.12688%2Fwellcomeopenres.15889.2 32656368 10.12688/wellcomeopenres.15889.1 PMC7327724

[3] Gan WH, Lim JW, Koh D. Preventing Intra-hospital Infection and Transmission of Coronavirus Disease 2019 in Health-care Workers. Safety and Health at Work. 2020;11(2):241–3. 10.1016/j.shaw.2020.03.001 32292622 PMC7102575

[4] Iacobucci G. Covid-19: Doctors sound alarm over hospital transmissions. BMJ. 2020;369:m2013. 10.1136/bmj.m2013 32430304

[5] Bandyopadhyay S, Baticulon RE, Kadhum M, Alser M, Ojuka DK, Badereddin Y, et al. Infection and mortality of healthcare workers worldwide from COVID-19: a scoping review. medRxiv. 2020:2020.06.04.20119594. 10.1101/2020.06.04.20119594 PMC7722361 33277297

[6] Zhan M, Qin Y, Xue X, Zhu S. Death from Covid-19 of 23 Health Care Workers in China. New England Journal of Medicine. 2020;382(23):2267–8. 10.1056/NEJMc2005696 32294342 PMC7179960

[7] Chirico F, Nucera G, Magnavita N. COVID-19: Protecting Healthcare Workers is a priority. Infect Control Hosp Epidemiol. 2020;41(9):1117. 10.1017/ice.2020.148 32299519 PMC7198459

[8] Kursumovic E, Lennane S, Cook TM. Deaths in healthcare workers due to COVID-19: the need for robust data and analysis. Anaesthesia. 2020;75(8):989–92. 10.1111/anae.15116 32397005 PMC7272944

[9] Cook T, Kursumovic E, Lennane S. Exclusive: deaths of NHS staff from covid-19 analysed [updated 22 April 2020; cited on 16 December 2020]. Available from: https://www.hsj.co.uk/exclusive-deaths-of-nhs-staff-from-covid-19-analysed/7027471.article.

[10] International Council of Nurses. More than 600 nurses die from COVID-19 worldwide 2020. [updated 3 June 2020; cited on 16 December 2020]. Available from: https://www.icn.ch/news/more-600-nurses-die-covid-19-worldwide.

[11] Centers for Disease Control and Prevention. Managing Exposed Healthcare Workers [updated 19 November 2020; cited on 15 December 2020]. Available from: https://www.cdc.gov/coronavirus/2019-ncov/hcp/non-us-settings/public-health-management-hcw-exposed.html#ManagingHCWs.

[12] Carter C, ThiLanAnh N, Notter J. COVID-19 Disease: Perspectives in Low-and Middle-Income Countries. Clinics in Integrated Care. 2020:100005. 10.1016/j.intcar.2020.100005 PMCID: PMC7261656

[13] Munharo S, Nayupe S, Mbulaje P, Patel P, Banda C, Gacutno KJA, et al. Challenges of COVID-19 testing in low-middle income countries (LMICs): the case of Malawi. Journal of Laboratory and Precision Medicine. 2020;5. 10.21037/jlpm-20-84

[14] World Health Organization. COVID-19 situation overview in Malaysia. Situation report 1–23 April 2020 [Cited on 15 December 2020]. Available from: https://www.who.int/docs/default-source/wpro—documents/countries/malaysia/coronavirus-disease-(covid-19)-situation-reports-in-malaysia/situation-report-malaysia-23-april-2020-final.pdf?sfvrsn=22ad02ca_6.

[15] Ministry of Health Malaysia. Guidelines COVID-19 Management In Malaysia No. 5/2020 2020 [Cited on 15 December 2020]. Available from: http://covid-19.moh.gov.my/garis-panduan/garis-panduan-kkm.

[16] Sim BLH, Chidambaram SK, Wong XC, Pathmanathan MD, Peariasamy KM, Hor CP, et al. Clinical characteristics and risk factors for severe COVID-19 infections in Malaysia: A nationwide observational study. The Lancet Regional Health—Western Pacific. 2020;4. 10.1016/j.lanwpc.2020.100055 33521741 PMC7837062

[17] Mohd Salleh NA, Hairi NN, Nik Farid ND, Isahak M, Ahmad Zaki R, Said MA, et al. COVID-19 Surveillance in University Malaya Medical Centre (UMMC) Guidelines for surveillance of healthcare workers. 2020.

[18] Samsudin MF, Wan KS, editors. Guidelines on Surveillance System Database for Healthcare Workers in University Malaya Medical Centre. 2020.

[19] Kim HY. Statistical notes for clinical researchers: Chi-squared test and Fisher’s exact test. Restor Dent Endod. 2017;42(2):152–5. 10.5395/rde.2017.42.2.152 28503482 PMC5426219

[20] Kawana A, Teruya K, Kirikae T, Sekiguchi J, Kato Y, Kuroda E, et al. "Syndromic surveillance within a hospital" for the early detection of a nosocomial outbreak of acute respiratory infection. Jpn J Infect Dis. 2006;59(6):377–9. 17186956

[21] Lai X, Wang M, Qin C, Tan L, Ran L, Chen D, et al. Coronavirus Disease 2019 (COVID-2019) Infection Among Health Care Workers and Implications for Prevention Measures in a Tertiary Hospital in Wuhan, China. JAMA Network Open. 2020;3(5):e209666–e. https://dx.doi.org/10.1001%2Fjamanetworkopen.2020.9666 32437575 10.1001/jamanetworkopen.2020.9666 PMC7243089

[22] Centers for Disease Control and Prevention (CDC). Interim Clinical Guidance for Management of Patients with Confirmed Coronavirus Disease (COVID-19) 2020 [updated 3 November 2020; Cited on 15 December 2020]. Available from: https://www.cdc.gov/coronavirus/2019-ncov/hcp/clinical-guidance-management-patients.html.

[23] Kluytmans-van den Bergh MFQ, Buiting AGM, Pas SD, Bentvelsen RG, van den Bijllaardt W, van Oudheusden AJG, et al. Prevalence and Clinical Presentation of Health Care Workers With Symptoms of Coronavirus Disease 2019 in 2 Dutch Hospitals During an Early Phase of the Pandemic. JAMA Network Open. 2020;3(5):e209673–e. 10.1001/jamanetworkopen.2020.9673 32437576 PMC7243090

[24] Phelan AL, Katz R, Gostin LO. The Novel Coronavirus Originating in Wuhan, China: Challenges for Global Health Governance. JAMA. 2020;323(8):709–10. 10.1001/jama.2020.1097 31999307

[25] Worldometer. Covid-19 Coronavirus pandemic 2020 [updated 15 December 2020; Cited on 15 December 2020]. Available from: https://www.worldometers.info/coronavirus/.

[26] Lahner E, Dilaghi E, Prestigiacomo C, Alessio G, Marcellini L, Simmaco M, et al. Prevalence of Sars-Cov-2 Infection in Health Workers (HWs) and Diagnostic Test Performance: The Experience of a Teaching Hospital in Central Italy. International Journal of Environmental Research and Public Health. 2020;17(12):4417. https://dx.doi.org/10.3390%2Fijerph17124417 32575505 10.3390/ijerph17124417 PMC7345358

[27] Rivett L, Sridhar S, Sparkes D, Routledge M, Jones NK, Forrest S, et al. Screening of healthcare workers for SARS-CoV-2 highlights the role of asymptomatic carriage in COVID-19 transmission. Elife. 2020;9:e58728. 10.7554/eLife.58728 32392129 PMC7314537

[28] Centers for Disease Control and Prevention. Interim Guidance on Testing Healthcare Personnel for SARS-CoV-2 [updated 14 December 2020; cited on 21 December 2020]. Available from: https://www.cdc.gov/coronavirus/2019-ncov/hcp/testing-healthcare-personnel.html.

[29] Willan J, King AJ, Jeffery K, Bienz N. Challenges for NHS hospitals during covid-19 epidemic. BMJ. 2020;368:m1117. 10.1136/bmj.m1117 32198166

[30] Aghaizu A, Elam G, Ncube F, Thomson G, Szilágyi E, Eckmanns T, et al. Preventing the next ’SARS’—European healthcare workers’ attitudes towards monitoring their health for the surveillance of newly emerging infections: qualitative study. BMC Public Health. 2011;11(1):541. 10.1186/1471-2458-11-541 21740552 PMC3160373

[31] Wee LE, Sim XYJ, Conceicao EP, Aung MK, Goh JQ, Yeo DWT, et al. Containment of COVID-19 cases among healthcare workers: The role of surveillance, early detection, and outbreak management. Infection Control & Hospital Epidemiology. 2020:1–7. 10.1017/ice.2020.219 32391746 PMC7248595

[32] Centers for Disease Control and Prevention. Scientific Brief: SARS-CoV-2 and Potential Airborne Transmission 2020 [updated 5 October 2020; cited on 24 February 2021]. Available from: https://www.cdc.gov/coronavirus/2019-ncov/more/scientific-brief-sars-cov-2.html

