# Correction: Implementation of a COVID-19 surveillance programme for healthcare workers in a teaching hospital in an upper-middle-income country

**DOI:** 10.1371/journal.pone.0268492

**Published:** 2022-05-11

**Authors:** Kim Sui Wan, Peter Seah Keng Tok, Kishwen Kanna Yoga Ratnam, Nuraini Aziz, Marzuki Isahak, Rafdzah Ahmad Zaki, Nik Daliana Nik Farid, Noran Naqiah Hairi, Victor Chee Wai Hoe, Sanjay Rampal, Chiu-Wan Ng, Mohd Fauzy Samsudin, Vinura Venugopal, Mohammad Asyraf, Narisa Hatun Damanhuri, Sanpagavalli Doraimuthu, Catherine Thamarai Arumugam, Thaneswaran Marthammuthu, Fathhullah Azmie Nawawi, Faiz Baharudin, Diane Woei Quan Chong, Vivek Jason Jayaraj, Venna Magarita, Sasheela Ponnampalavanar, Nazirah Hasnan, Adeeba Kamarulzaman, Mas Ayu Said

Victor Chee Wai Hoe is not included in the author byline. Victor Chee Wai Hoe should be listed as the ninth author, and his affiliation is 1: Centre for Epidemiology and Evidence-based Practice, Department of Social and Preventive Medicine, Faculty of Medicine, University of Malaya, Kuala Lumpur, Malaysia. The contributions of this author are as follows: Conceived and designed the experiments, contributed reagents/materials/analysis tools, and approved the final version to be published.
