# Supplementary material for: Implementation of a COVID-19 surveillance programme for healthcare workers in a teaching hospital in an upper-middle-income country
Source: PLoS One. 2021 Apr 14;16(4):e0249394. doi: 10.1371/journal.pone.0249394 (PMC8046251; doi:10.1371/journal.pone.0249394)
Supplement: S1 Appendix — (DOCX) [file pone.0249394.s001.docx]

**S1 Appendix: Assessment checklist for healthcare workers (HCW) who were exposed to COVID-19 patients**

| NAME | | | : | STAFF NO | | : | | | |
| --- | --- | --- | --- | --- | --- | --- | --- | --- | --- |
| PHONE NO | | | : | DEPARTMENT | | : | | | |
| POSITION | | | : | IC NO | | : | | | |
| ADDRESS | | |  | | | | | | |
| Assessment No: | | | : | | | | | | |
|  |  | | | | | | | | |
| 1. | Are you exposed to suspected or suspected patients COVID-19?  Patient name: ______________________________________  Date of exposure: ___________________________________ | | | | YES | |  | NO |  |
| 2. | If YES, was the patient wearing a surgical mask? | | | | YES | |  | NO |  |
|  | Distance: | |  | | | | | | |
|  | Duration: | |  | | | | | | |
|  |  |  | | | | | | | |
| 3. | Do you engage in an investigation related to a physical or COVID-19 patient? | | | | YES | |  | NO |  |
| 4. | If YES, state the method of exposure, procedure: | | | | | | | | |
| 5. | If you are wearing personal protective equipment (PPE), specify the PPE that you used. | | | | | | | | |
|  | 1. Mask? (Surgical mask / N95) | | | | YES | |  | NO |  |
|  | 1. Face shield? | | | | YES | |  | NO |  |
|  | 1. Glove? | | | | YES | |  | NO |  |
|  | 1. Gown? | | | | YES | |  | NO |  |
| 6. | Do you follow PPE Protocol (Doffing and Donning steps) | | | | YES | |  | NO |  |
|  |  | | | | | | | | |
| 7. | Do you do a hand wash/Hand rub (e.g.: after remove PPE, after meeting COVID-19 patient)? | | | | YES | |  | NO |  |
| 8. | Are COVID-19 patients cough or sneezing in front of your face? | | | | YES | |  | NO |  |
| 9. | Do you currently have any these symptoms, cough, myalgia, arthralgia, GI symptoms, hard breathing, sore throat and fever? Specify. | | | | YES | |  | NO |  |
|  |  | | | | | | | | |
| 10. | Do you have any underlying comorbidity? _______ | | | |  |  |  |  |  |

11. Diagnosis: _________________________

12. Plan:

Date of 1^st^ Swab: ______________

Date of 2^nd^ swab: ­­­­­­­­­­­­­­­­­______________

Date of Home Surveillance: from ___________ to __________ (Total days: ___________)

Date of Return to work OSHE clinic Appointment:

Keen for Psychosocial support *circle: YES/NO
