# Supplementary material for: Implementation of a COVID-19 surveillance programme for healthcare workers in a teaching hospital in an upper-middle-income country
Source: PLoS One. 2021 Apr 14;16(4):e0249394. doi: 10.1371/journal.pone.0249394 (PMC8046251; doi:10.1371/journal.pone.0249394)
Supplement: S2 Appendix — (PDF) [file pone.0249394.s002.pdf]

S2 Appendix

UNIVERSITY OF MALAYA MEDICAL CENTRE

PPE Recommendations

IMPORTANT!

ASSESS THE RISK of exposure to body substances or contaminated surfaces BEFORE any health care activity.
Select PPE based on the assessment of risk.
Please make sure all the patients are wearing their masks.
Maintain spatial distance of more than 1m at all times when possible.

Please note that the sets below illustrate common clinical examples, and the list is not exhaustive.
The sets below do not include PPE for the operating theatre.
Please consult the infection control team (+6012 691 4556) should you have any doubts/ queries.

Set of PPE	COVID-19 Related (Confirmed or Suspected COVID-19/ SARI/ ILI/PUS)	All Other Patients
**Set 1A**<br>**All Staff in Hospital Premises**<br>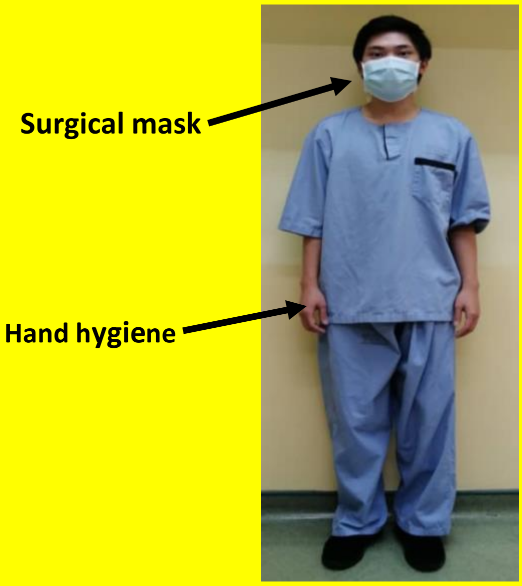	**Not applicable**	**Areas and Activities:**<br>• **Front desk, triage counter and dispensing medication at general pharmacies**<br>○ **If there is a physical barrier (e.g.: glass or plastic window), eye protection is not needed**<br>• **Ward rounds, consultation room***<br><br>*** Face shield/goggle should be used:**<br>○ **Based on risk assessment**<br>○ **If a patient unable to wear a mask**<br>○ **Conducting oropharyngeal examination**

By Infection Control Department & Medical Development Unit, UMMC
Updated 10/2/2021 -approved by UMMC COVID Task Force Committee

**Set 1B**<br>**All Staff in Hospital Premises**<br>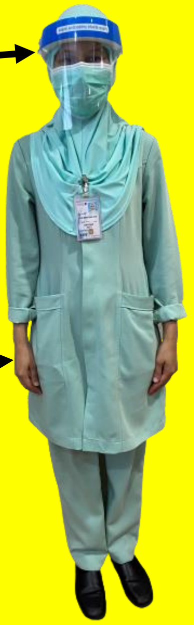 	**Areas and Activities:**<br>• Front desk<br>• Triage counter at RUKA, ED, SSC	• Front desk and triage counter<br>○ If there is no physical barrier (glass or plastic window)<br>• When attending to patients who are unable to wear a mask<br>• When performing oropharyngeal examination
**Set 2A**<br>**Standard Precaution (Aseptic)**<br>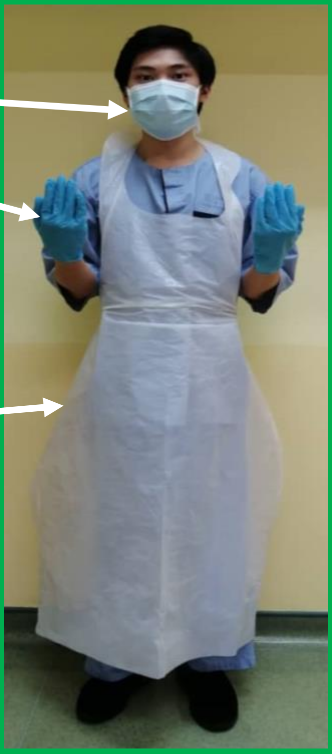<br><br>• Face shield/goggle should be used based on risk assessment	Not applicable	Examples of activities:<br>• Blood taking [1]<br>• All intravenous line insertions[2]<br>• Blood culture [2]<br><br>[1] If splash contamination is anticipated, to wear long sleeved plastic apron and/or face shield<br><br>[2] To use sterile gloves<br><br>Eye protection shoud be used:<br>• Based on risk assessment<br>• If a patient unable to wear a mask<br>• Conducting oropharyngeal examination

Set 2B
Standard Precaution (Sterile)

Surgical mask

Sterile gloves

Long sleeved sterile gown

Hand hygiene

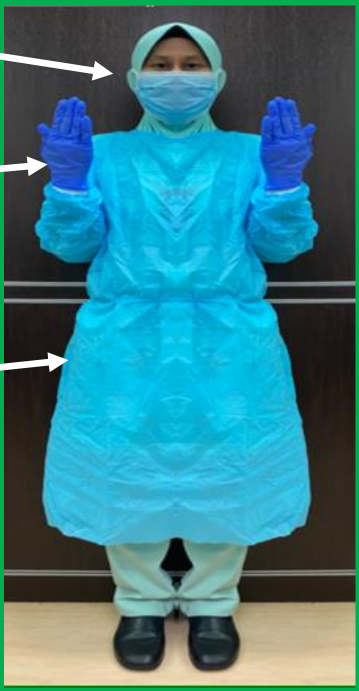

- Face shield/goggle should be used based on risk assessment

Not applicable

Examples of sterile procedures:

- Neuraxial anasthesia
- Peripheral nerve block
- Local anaesthesia
- Field blocks
- CVL and artline insertion/
- Removal
- Interventional radiology procedures e.g. thrombectomy, image-guided biopsies
- Angiogram/ Angioplasty

*Eye protection shoud be used:

- Based on risk assessment
- If a patient unable to wear a mask
- Conducting oropharyngeal examination

Set 3
Standard & Additional Precaution

Face shield[4]

Surgical mask

Long sleeved plastic apron/ gown

Hand hygiene

Gloves

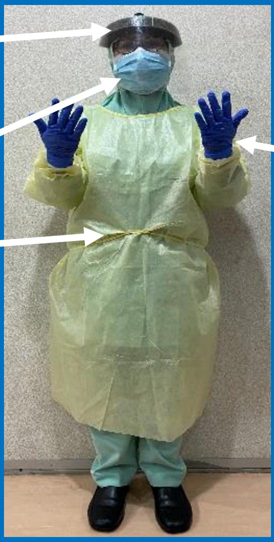

[4] For extended use:

- N95 mask and face shield
- To use gown together with plastic apron on top and to change plastic apron and gloves between patient

Low Risk [3]

Activities:

- Direct routine care of low-risk patient
- Blood taking
- Clerking and vital signs monitoring
- Cleaning of patient's room
- Escorting patient (within 1m distance of patient)

[3] Low risk:

- Patient is not actively coughing
- Asymptomatic
- Patient is able to wear a mask

PPE use i.e.,
- N95 mask/surgical face mask
- ±eye/face protection
- ± long sleeved apron/isolation gown
- ± gloves

is based on the risk assessment done by the INDIVIDUAL healthcare worker, which is determined by the risk of contamination/splashes during the activity/procedure and the diagnosis of the patient)

Examples of activities:
- Care giving activities with risk of contamination or splash: i.e., Toileting care

By Infection Control Department & Medical Development Unit, UMMC
Updated 10/2/2021 -approved by UMMC COVID Task Force Committee

Set 4

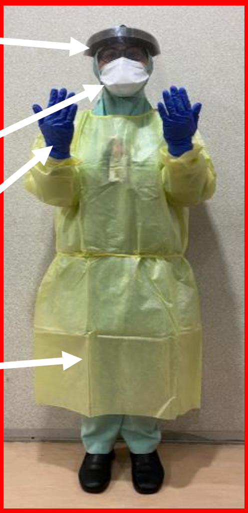

[4] For extended use:

- N95 mask and face shield
- To use gown together with plastic apron on top and to change plastic apron and gloves between patient

High Risk [6]

Activities:

- Direct routine care of high risk patient
- Performing and assisting nasopharyngeal swab

[6] High risk:

- Patient is coughing
- Patient requires oxygen support
- Patient unable to wear surgical mask
- Ventilated patient

Examples aerosol generating procedures/activities:

- Administration of medication via nebulization
- Obtaining nose and throat swabs
- Administration of pressurized humidified oxygen
- High flow nasal oxygenation
- Non-invasive ventilation e.g., BiPAP and CPAP
- Suctioning
- Sputum induction
- Chest physiotherapy
- Nasogastric tube insertion and feeding
- Clinical dysphagia examination
  - this examination includes orofacial assessment and administration of food and/or fluids to evaluate swallowing ability
- Lower GI endoscopy
- Laser plume
- Active breathing circuit (Oncology)
- Intubation and extubation
- Manual ventilation
- Cardiopulmonary resuscitation
- Bronchoscopy
- Surgery with high speed devices used
- Post mortem procedures in which high-speed devices are used
- Dental procedures
- Front of neck airway procedures e.g.

		tracheostomy, cricothyroidotomy<br>• ENT procedures (e.g. laryngoscopy, FEES)<br>• Upper GI endoscopy<br>• Transoesophageal ECHO
**Set 5**<br>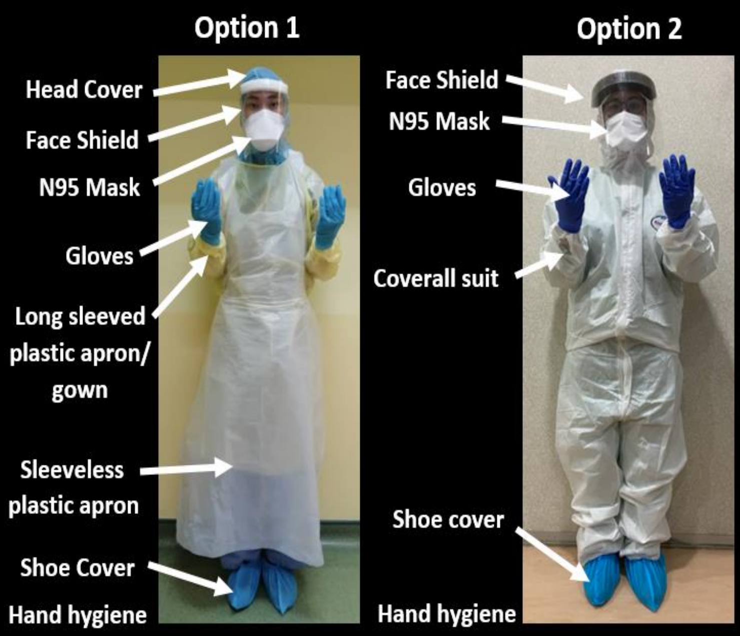<br>+ Alternatively, PAPR can be used	Examples of procedures:<br>• Tracheostomy<br>• Intubation and extubation<br>• Manual ventilation<br>• Suctioning<br>• Bronchoscopy<br>• Cardiopulmonary resuscitation<br>• Nebulisation<br>• Non-invasive ventilation (NIV) e.g. BiPAP and CPAP<br>• Surgery [7] and post mortem procedures in which high-speed devices are used<br>• High-flow nasal oxygen (HFNO)<br>• Induction of sputum<br>• Dental procedures<br>[7] For detailed PPE for surgery and sterile procedures, please refer to Annex 14 of UMMC COVID-19 guidelines	Not applicable
