# Supplementary figures and images for: Implementation of a COVID-19 surveillance programme for healthcare workers in a teaching hospital in an upper-middle-income country
Source: PLoS One. 2021 Apr 14;16(4):e0249394. doi: 10.1371/journal.pone.0249394 (PMC8046251; doi:10.1371/journal.pone.0249394)

**S3 Appendix: Management of exposed HCW categorised as high-risk**

**
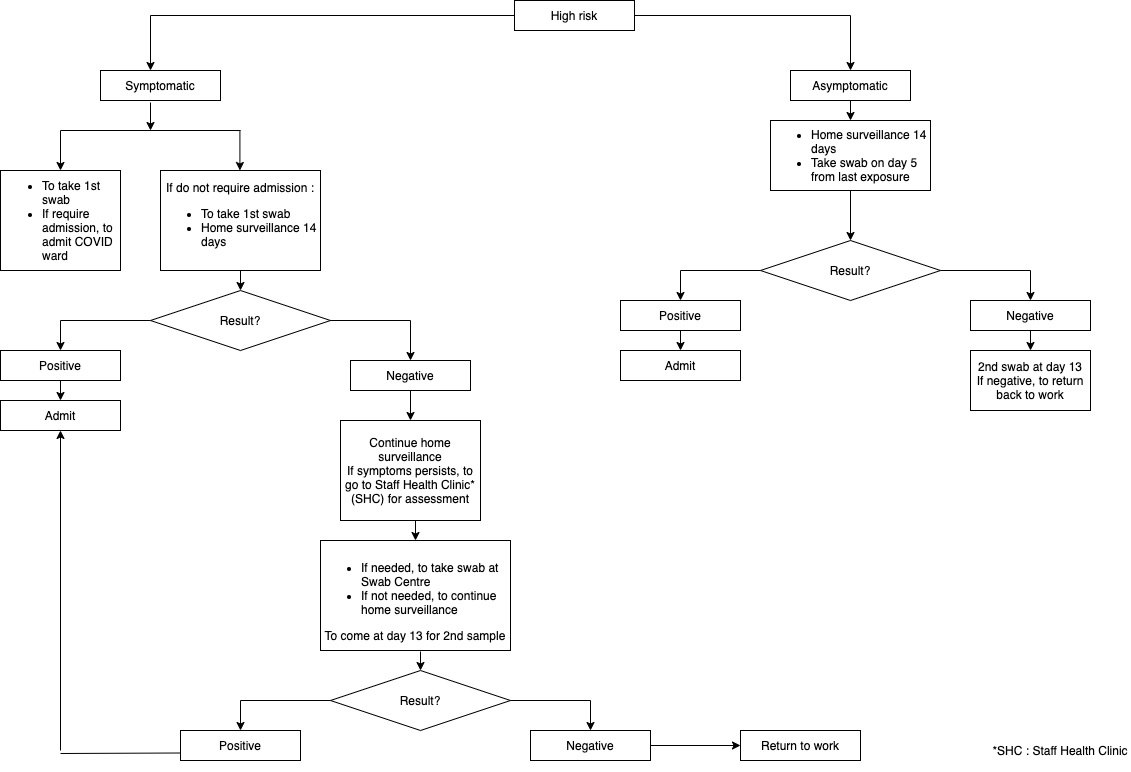
**

Supplement: S3 Appendix — (DOCX) [file pone.0249394.s003.docx]

**S4 Appendix: Management of exposed HCW categorised as medium-risk**


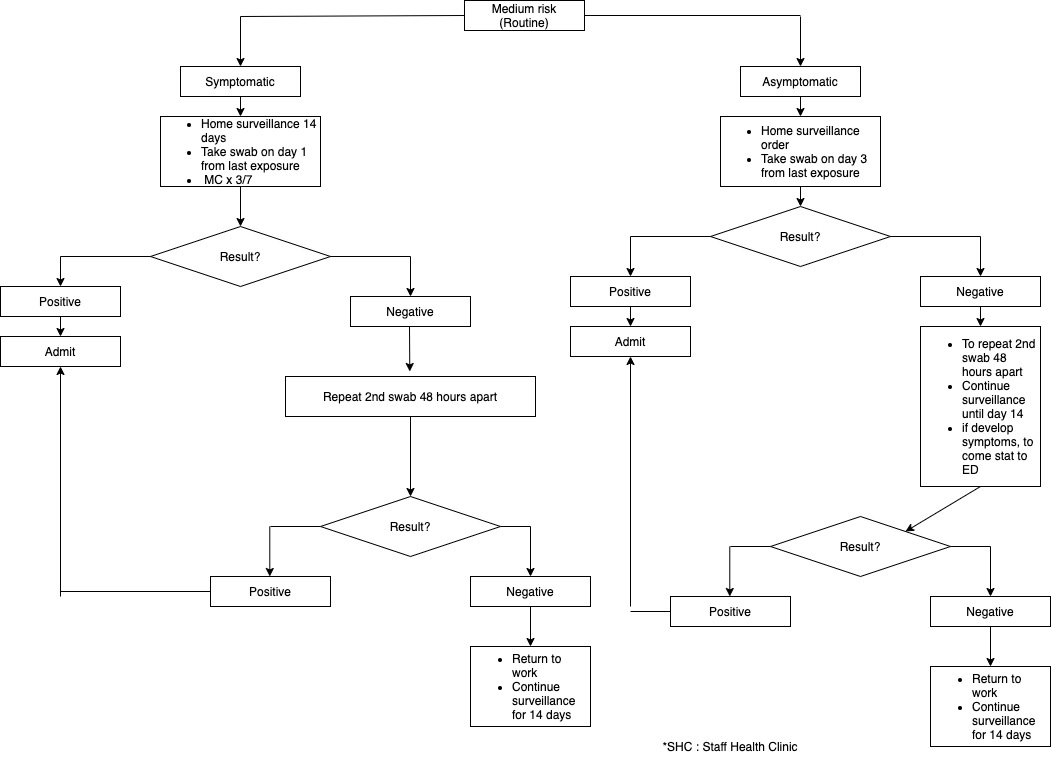

Supplement: S4 Appendix — (DOCX) [file pone.0249394.s004.docx]
