# Supplementary material for: Implementation of a COVID-19 surveillance programme for healthcare workers in a teaching hospital in an upper-middle-income country
Source: PLoS One. 2021 Apr 14;16(4):e0249394. doi: 10.1371/journal.pone.0249394 (PMC8046251; doi:10.1371/journal.pone.0249394)
Supplement: S5 Appendix — (DOCX) [file pone.0249394.s005.docx]

**S5 Appendix: Management of exposed HCW categorized as low-risk**

a) Symptomatic

• Take swab immediately (Day 1 of exposure).

• If swab is positive, for admission.

• If first swab is negative, to continue work with surgical masks and frequent hand-hygiene.

• If symptom persist or worsen or occur at any day during the 14 days of surveillance, to go to special Staff Health Clinic in the Department of Primary Care Medicine during office hours or the Emergency Department during non-office hours for further investigation and management with surgical masks.

• Home surveillance letter from the Occupational Safety Health and Environment (OSHE) unit for 2 days while waiting for the result.

• Surveillance under surveillance team for 14 days from day of exposure.

b) Asymptomatic

• No work restrictions

• Surveillance under surveillance team for 14 days from day of exposure.

• If symptoms developed (detected by surveillance team), for review in Special Staff Health Clinic in the Department of Primary Care Medicine and schedule for swab in Swab centre.
