# Supplementary material for: Implementation of a COVID-19 surveillance programme for healthcare workers in a teaching hospital in an upper-middle-income country
Source: PLoS One. 2021 Apr 14;16(4):e0249394. doi: 10.1371/journal.pone.0249394 (PMC8046251; doi:10.1371/journal.pone.0249394)
Supplement: S6 Appendix — (PDF) [file pone.0249394.s006.pdf]

S6 Appendix

UMMC Portal (*Portal Staf* MyUMMC) COVID-19 Healthcare Worker Risk Assessment and Surveillance

The COVID-19 Healthcare Worker Risk Assessment and Surveillance functions in the UMMC Portal (*Portal Staf* MyUMMC) were created in collaboration with the UMMC Department of Information Technology (*Jabatan Teknologi Maklumat*, JTM). A special ID was created for the members of Surveillance Operation Room to gain access to the data in the portal as system admin for the COVID-19 Healthcare Worker Risk Assessment & Surveillance page.

1.1 Main page

Once logged in using the specifically allocated ID, the portal will bring user to the main page as shown in the **Figure 1** below. In addition to the HCW Risk Assessment / Surveillance function, which is also available for all UMMC Staff, the system administrator has access to the reports and list of feedback for both Risk Assessment and Surveillance of PUS (HCW).

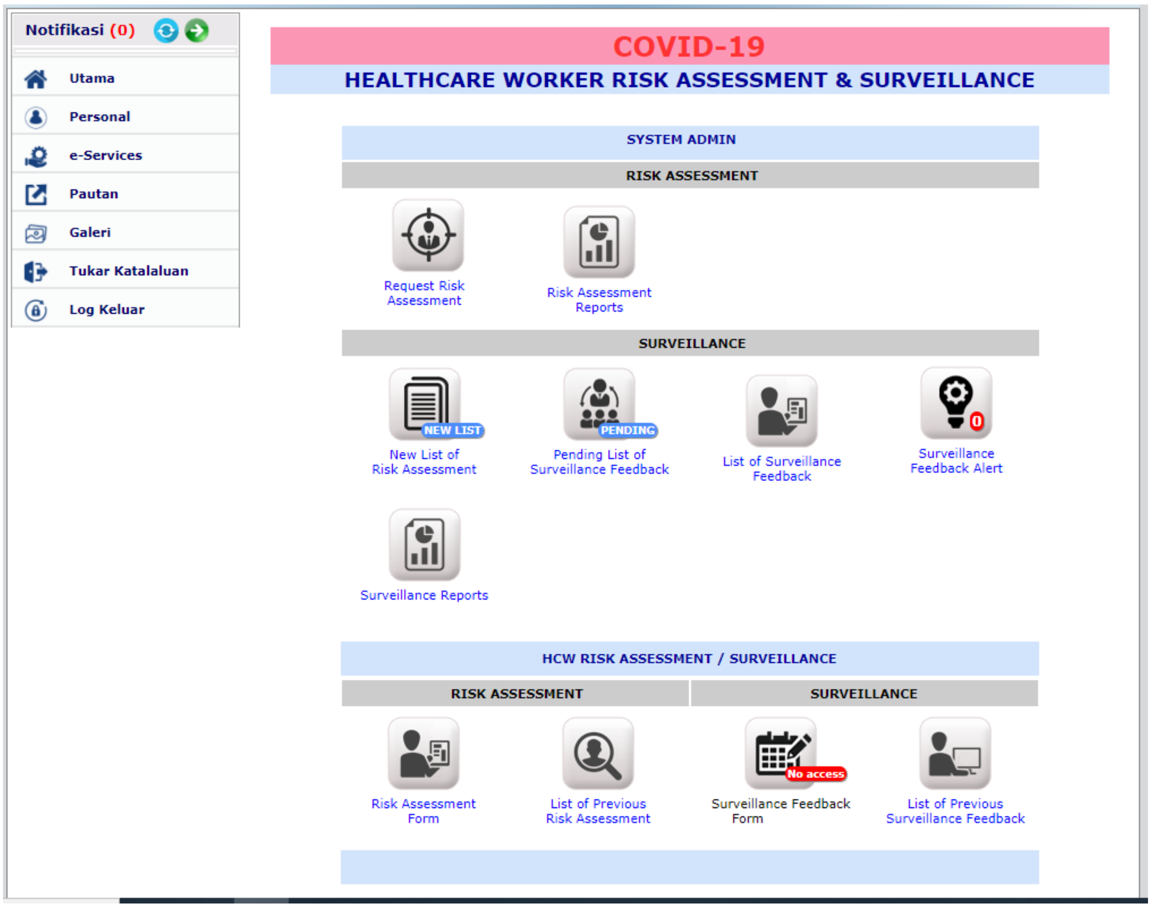

Figure 1: COVID-19 Healthcare Worker Risk Assessment & Surveillance

1.2 System Admin: Risk Assessment

1.2.1 Request Risk Assessment

This function is for the Risk Assessment team members to send an email and SMS to the healthcare workers exposed to COVID-19 patients, to remind them to fill in the Risk Assessment form in the portal.

1.2.2 Risk Assessment Report

User can access the report of risk assessment submitted by the healthcare workers from this function by clicking on the button which will bring the user to the page as illustrated in **Figure 2**:

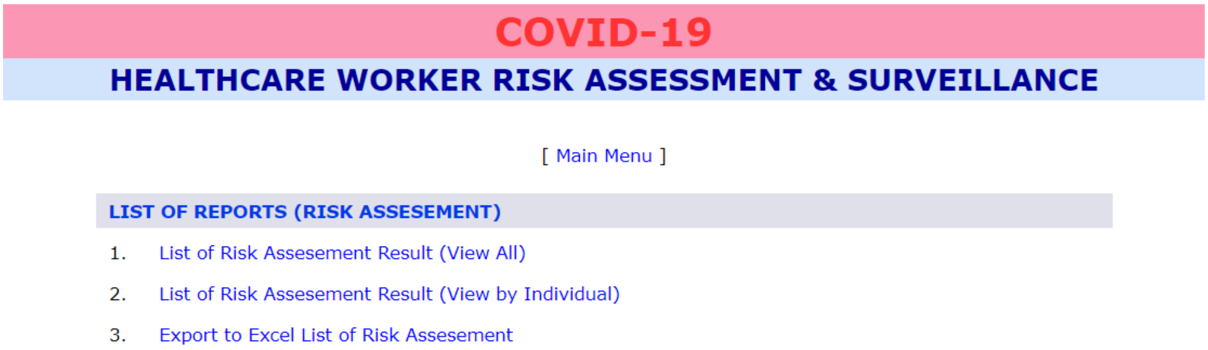

Figure 2: List of Reports (Risk Assessment)

To view the individual risk assessment results, click on the "List of Risk Assessment Result (View by Individual)". User will be brought to the list as shown below in **Figure 3**. User can also search for the risk assessment of the HCW in by their name, Staff ID, or I/C number.

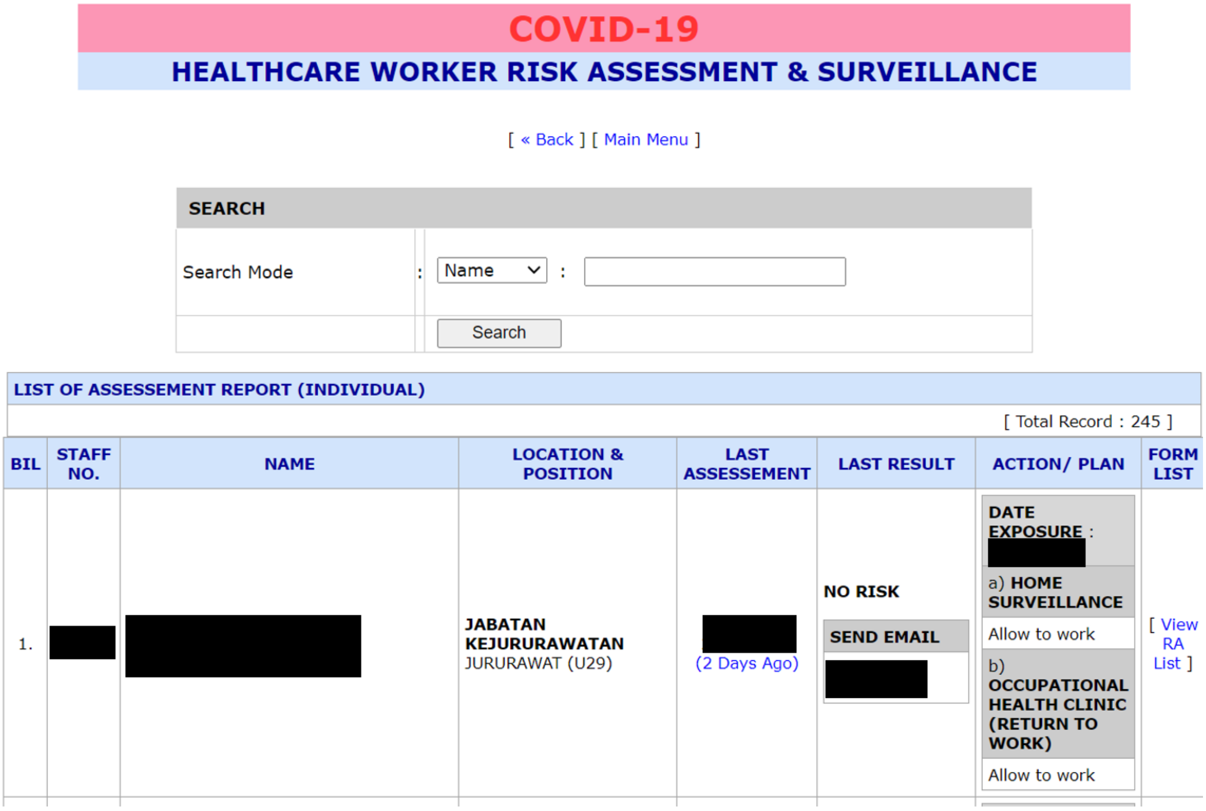

Figure 3: List of Assessment Report (Individual)

User can also view the list of risk assessments (RAs) conducted for individual HCW by clicking at “View RA list” at the last column under “FORM LIST”. The RA list can also be accessed from the “List of HCW Surveillance Feedback (Individual)” which will be explained in later part of this guideline. Each individual RA form can be accessed from the RA list to obtain further information on the details of the exposures and risks.

1.3 System Admin: Surveillance

Under the Surveillance section, there are five functions that can be accessed by user, namely: *“New List of Risk Assessment”, “Pending List of Surveillance Feedback”, “List of Surveillance Feedback”, “Surveillance Feedback Alert”,* and *“Surveillance Reports”.*

1.3.1 New List of Risk Assessment

This function will alert the user on the recent self risk assessment submitted by HCW through the portal. By default, only risk assessment within 24 hours and other than “No Risk” status will appear on the list. For other specific action, user would have to use the filter provided.

User will click on the Send SMS button to send reminder to the PUS to fill in the daily surveillance feedback. PUS (HCW) will receive the SMS immediately once the button is clicked. For the subsequent days of surveillance, the system will send the SMS automatically on daily basis at 6 a.m. A second reminder message will follow at 10 a.m. for those who are yet to fill the surveillance feedback for the day. The final reminder will be sent at 12 p.m.

1.3.2 Pending List of Surveillance Feedback

This function allows user to view the list of PUS (HCW) who are yet to submit their surveillance feedback for the day.

1.3.3 List of Surveillance Feedback

This function allows user to view the list of PUS (HCW) currently under active surveillance. User will have to click on discharge button once the surveillance of the PUS (HCW) is completed.

1.3.4 Surveillance Feedback Alert

This function will alert user for any PUS (HCW) reporting any new or worsening symptoms based on the 24-hour surveillance feedback.

1.3.5 Surveillance Reports

This function provides user with a list of reports for surveillance activity including the list of HCW surveillance feedback, surveillance statistics, surveillance discharge list, and the list of PUS (HCW) with alert (or based on previous feedback). User can also export the list of surveillance feedback to Microsoft Excel form, which can be modified to create the daily line-listing to be submitted to the PKD.

User can view individual surveillance feedback from here by clicking at the “List HCW Surveillance Feedback (View by Individual)”, which will bring them to the page as illustrated in the following **Figure 4**:

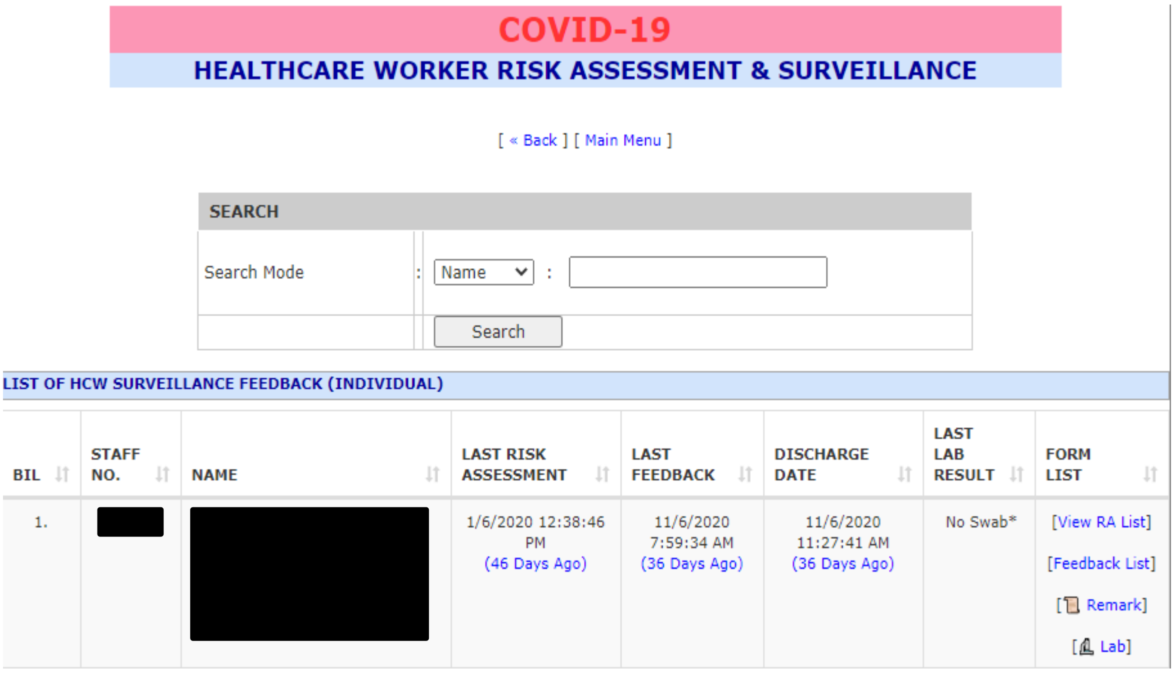

Figure 4: List of HCW Surveillance Feedback (Individual)

In this page, user will `be able to search for specific PUS (HCW) by using either name, Staff ID, or I/C number. List of RAs, list of daily surveillance feedback(s), and laboratory result(s) for the specific PUS can also be accessed by clicking on the respective links located in the last column under “FORM LIST”. In addition, remarks can be made by user to document any additional information for the specific PUS, which can be accessed under the same column.

By clicking on the Feedback List, user can gain access to the details of symptoms feedback reported by the PUS for data entry into DETECT system.
